# Similar outcomes in computer-assisted and conventional total knee arthroplasty: ten-year results of a prospective randomized study

**DOI:** 10.1186/s12891-021-04556-3

**Published:** 2021-08-18

**Authors:** Franziska Beyer, Alexander Pape, Cornelia Lützner, Stephan Kirschner, Jörg Lützner

**Affiliations:** 1 University Hospital Carl Gustav Carus, TU Dresden, Fetscherstr. 74, 01307 Dresden, Germany; 2 St. Vincentius-Kliniken, ViDia Christliche Kliniken Karlsruhe, Steinhäuserstraße 18, 76135 Karlsruhe, Germany

**Keywords:** Knee arthroplasty, Knee replacement, Navigation, Computer-assisted, Results, Patient-reported outcome

## Abstract

**Background:**

Computer-assisted navigation (CAS) was developed to improve the surgical accuracy and precision. Many studies demonstrated better alignment in the coronal plane in CAS TKA compared to conventional technique. The influence on the functional outcome is still unclear. Only few studies report long-term results of CAS TKA. This study was initiated to investigate 10-year patient-reported outcome of CAS and conventional TKA.

**Methods:**

From initially 80 patients of a randomized study of CAS and conventional TKA a total of 50 patients could be evaluated at the 10-year follow-up. The Knee Society Score and EuroQuol Questionnaire were assessed. For all patients a competing risk analysis for revision was performed.

**Results:**

The patient-reported outcome measures demonstrated similar values for both groups. The 10-year risk for revision was 2.5% for conventional TKA and 7.5% for CAS TKA (p=0.237).

**Conclusions:**

There was no difference between CAS and conventional TKA with regard to patient-reported outcome and revision risk ten years after surgery.

**Trial registration:**

This study was registered at clinicaltrials.gov on 11/30/2009, ID: NCT01022099.

## Background

Total knee arthroplasty (TKA) is a very effective treatment option for end-stage ostearthritis of the knee. The influence of knee alignment on outcome and revision rates after TKA has been debated controversially. While some studies demonstrated an increased revision risk in malaligned TKA [1, 2] other studies did not find a difference between TKA with a mechanical axis within or outside 0 ± 3° [3–5]. Furthermore, since the concepts of constitutional varus [6] and kinematic alignment [7] were introduced a neutral leg alignment is not always desired. Although the ideal leg axis is still under discussion, the individually planned alignment should be achieved and a relevant malalignment should be avoided [8, 9].

Computer-assisted navigation (CAS) was developed to improve the surgical accuracy and precision. In systematic reviews, many studies demonstrated better alignment in the coronal plane in CAS TKA compared to conventional technique [10, 11] but did not improve rotational alignment [12]. It has been expected that this improved accuracy will result in better patient-reported outcome measures (PROMs) and reduced revision rates. Studies investigating mid-term PRO demonstrated mostly similar results between CAS and conventional TKA. Only few studies report long-term results of CAS TKA. Some of these studies demonstrated better long-term survival of CAS TKA [13–16]. Additional advantages of CAS TKA are the more accurate and more effective soft-tissue balancing due to the direct response from the CAS system. Furthermore, CAS is a valuable measuring and teaching tool. Disadvantages of computer navigation include increased costs, longer operating time, the risk of fractures around pin sites and pin site infection. However, the overall risk of CAS-specific complications has been described as very low [17]. To date the role of CAS TKA is still under debate [18].

This study was initiated to investigate the long-term patient-reported outcome of CAS and conventional TKA. We hypothesized better PROMs in CAS TKA.

## Methods

This is a follow up study on a previously puplished prospective randomized clinical trial [19–21].A total of 80 patients scheduled for TKA between January 2006 and April 2007 were randomized to CAS or conventional surgical technique after informed consent. All patients were operated by two surgeons experienced in both techniques, conventional and CAS TKA (SK, JL). Both surgeons have performed at least 30 CAS TKA before the study. All patients received the same cemented, unconstrained, cruciate-retaining TKA with a rotating platform (Scorpio PCS, Stryker, Mahwah, NJ, USA) without patellar resurfacing. All surgeries were performed using a medial patellofemoral approach and a femur-first measured resection technique. Soft-tisue balancing was performed after the bone cuts. All surgeries aimed at a neutral leg axis (mechanical alignment). In the CAS group an imageless navigation system was used (Stryker navigation, Stryker, Mahwah, NJ).

Patients were seen by a trained study nurse before surgery, at 2, 5 and 10 years and knee function and health-related quality of life (HrQoL) were obtained using the Knee Society Score (KSS) and the EuroQuol questionnaire (EQ-5D). The KSS is divided into a Knee Score and a Function Score. The Knee Score consists of items on pain, range of motion, stability and alignment of the leg, the Function Score includes information on walking distance, stair climbing and walking aids. In both scores a total of 100 points indicates full function. The EQ-5D describes the self-perceived health state of the patient using subgroups of mobility, self-care, usual activities, pain/discomfort, anxiety/depression. The subgroups are divided into three levels (no problems, some problems, serious problems/unable to perform). From these answers an index can be calculated in which 0 indicates the worst and 1.0 the best HrQoL. Additionally, a visual analogue scale (VAS) records the patient`s current health state. A value of 100 indicates the best and a value of 0 indicates the worst imaginable health state.

The study protocol for the long-term follow-up was approved by the local independent ethics committee in March, 2011 (EK6012011). Study follow-up was completed in May 2017 as the study ended regularly.

### Statistical analysis

Endpoints of this investigation were differences between the two groups in knee function (Knee Society Score) or in self-perceived general health state (EQ-5D). Due to low case number and not normally distributed data, description was based on medians and inter-quartile ranges for continuous values and on absolute and relative frequencies for categorical endpoints, respectively. Comparisons between groups were based on two sample Wilcoxon tests for continuous endpoints and on Fisher`s exact tests for categorical endpoints, respectively. Results of these exploratory significance tests were summarized in p-values, where p<0.05 indicates statistically significant differences between groups. All analyses were performed using SPSS® software (release 24.0 for Windows®, IBM Corp, Armonk, NY).

To estimate the risk of revision, the cumulative incidence function, which takes account of death as a competing risk, was used. Survival data were analysed using the statistical software R (R Foundation for Statistical Computing, Vienna, Austria).

## Results

**Fig. 1:**
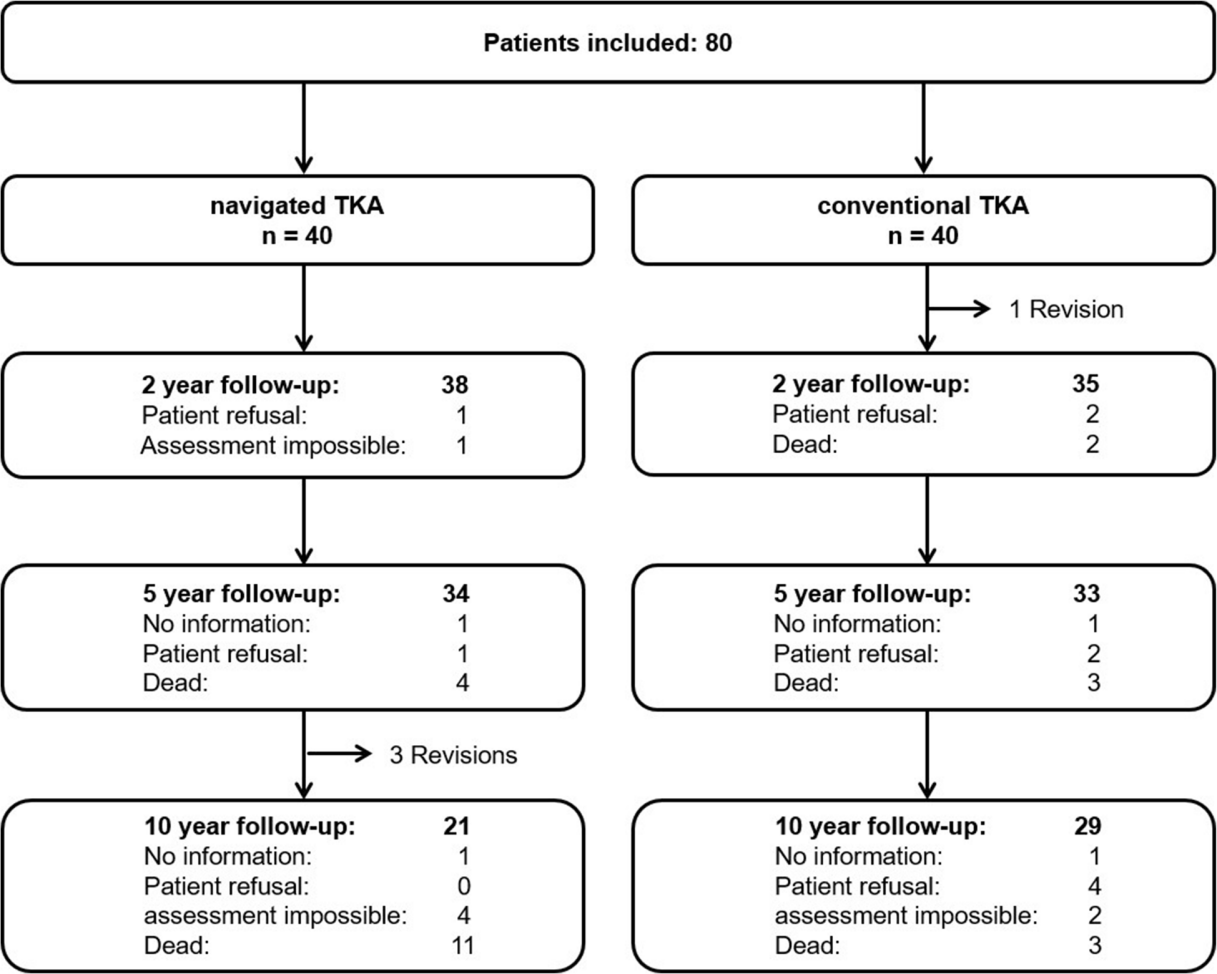
Flow-Chart of study patients

Fourteen patients died before the 10-year follow-up. Twelve of the remaining patients did not return for a follow-up visit: two patients could not be contacted, six were unable to attend the follow-up examination due to illness unrelated to the knee arthroplasty and four refused to attend (Fig. 1).

There were fewer outliers outside a range of 3° from a neutral leg axis in the navigated group, but this difference was not statistically significant. There were no statistically significant differences in coronal, sagittal or rotational alignment of the femoral and tibial components between the two groups [21].

Four patients had undergone revision: one patient from the conventional group due to a preoperatively unknown metallic hypersensitivity with persistent swelling one year after surgery and three patients of the CAS group (one each for aseptic loosening after eight years, instability after nine years and periprosthetic infection after nine years). The 10-year risk for revision (all causes) was 2.5% for conventional TKA and 7.5% for CAS TKA using competing risk survival analysis (p=0.237).

Further reoperations included one secondary patellar resurfacing four years after surgery, one periprosthetic femur fracture which was treated by open reduction and internal fixation (both in the conventional group) and two acute periprosthetic infections in the CAS group (5 and 7.5 years after primary surgery) which were successfully treated with debridement, irrigation, insert exchange and antibiotics (DAIR).

The remaining patients in both groups were still comparable at baseline for age, sex, comorbidities (ASA-Score) and body mass index (BMI, Table 1).

**Table 1:** Pre-operative data of the remaining 50 patients. Data are given as medians (25^th^, 75^th^ percentile)

	CAS TKA n=21	Conventional TKA n=29	p-value
**Pre-operative patient characteristics**			
Age at operation (years)	66 (61, 73)	69 (62, 75)	0.776
Sex (% female)	71.4	62.1	0.557
ASA (% 1+2)	71.4	51.7	0.243
BMI (kg/m^2^)	30.8 (27.0, 32.3)	29.6 (26.8, 33.1)	0.791
Cut-Sew-Time (min)	88 (82, 92)	83 (74, 88)	0.019

**Fig. 2:**
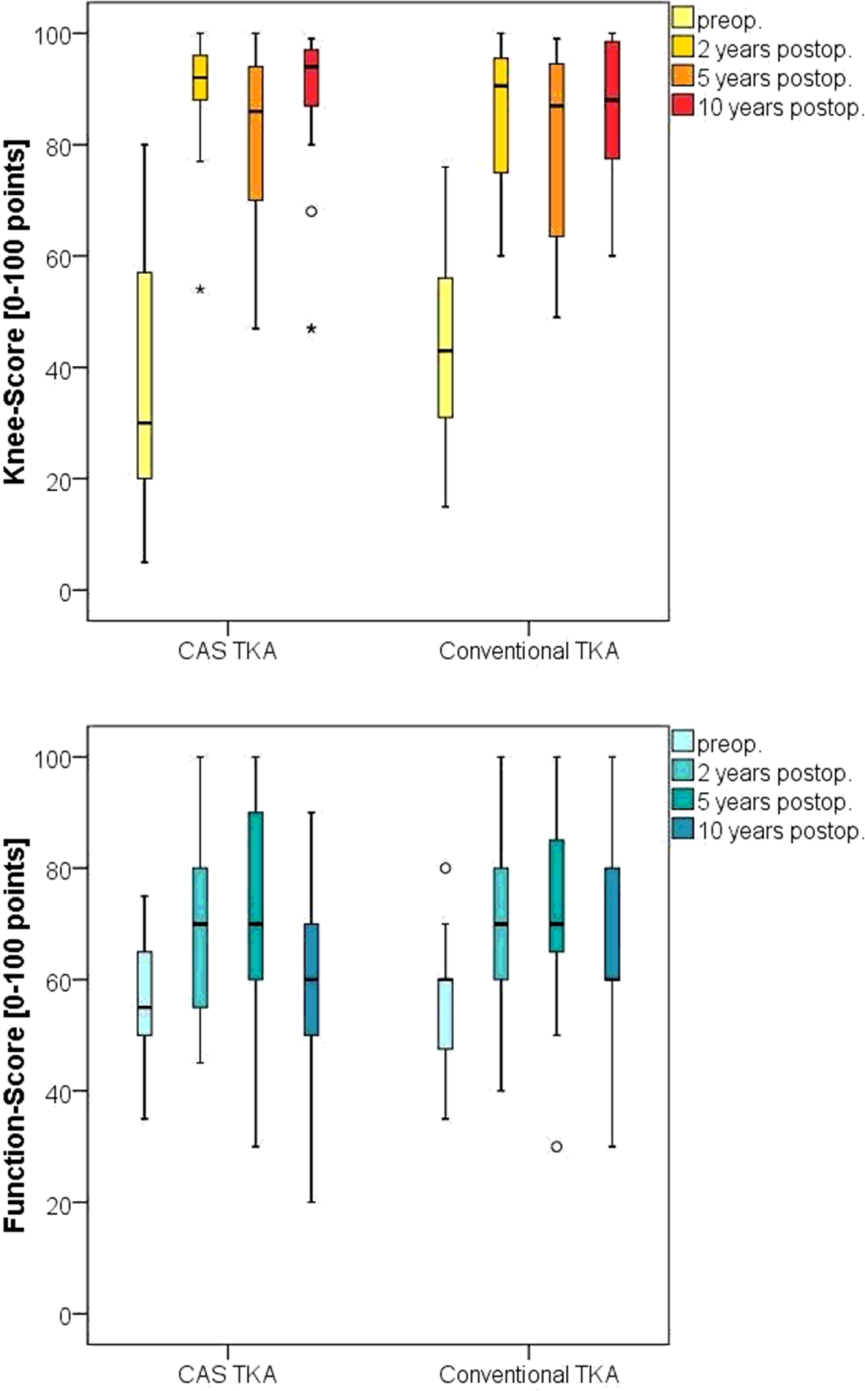
Knee Society Knee and Function Score, the line in the box represents the median, the box the 25^th^ to 75^th^ percentile, the whiskers represent the highest and lowest values which are no greater than 1.5 * interquartile range, circles and stars represent outliers

The *Knee Society Score* showed lower values pre-operatively in the navigated group and similar results to the conventional group at the 2, 5 and 10-year follow-up. For both groups no relevant decrease in the *Knee Score* could be noted, whereas the *Function Score* decreased after 5 years (Fig. 2)

Health related quality of life increased up to two years after TKA, then slightly decreased. No significant differences have been observed between the two groups (Table 2).

**Table 2:** Results of the Knee Society Score and EuroQol Questionnaire. Data are given as medians (25^th^, 75^th^ percentile)

	CAS TKA n=21	Conventional TKA n=29	p-value
**KSS Knee-Score [0 - 100]**			
before surgery	29 (20, 57)	42 (35, 53)	0.097
2 year follow-up	93 (89, 97)	93 (75, 99)	0.838
5 year follow-up	87 (69, 93)	88 (66, 95)	0.813
10 year follow-up	92 (74, 97)	88 (77, 99)	0.696
**KSS Function-Score [0 - 100]**			
before surgery	50 (45, 60)	58 (48, 60)	0.926
2 year follow-up	70 (55, 80)	70 (50, 80)	0.929
5 year follow-up	70 (60, 90)	70 (53, 80)	0.581
10 year follow-up	60 (50, 70)	60 (40, 70)	0.707
**EuroQol Visual Analogue Scale [0 - 100]**			
before surgery	50 (40, 50)	50 (40, 60)	0.146
2 years	70 (50, 80)	65 (50, 75)	0.545
5 years	80 (65, 80)	65 (50, 80)	0.200
10 years	50 (50, 75)	63 (45, 78)	0.951
**EuroQol Index [0 - 1]**			
before surgery	0.26 (0.17, 0.79)	0.70 (0.26, 0.79)	0.142
2 years	0.79 (0.79, 0.89)	0.89 (0.70, 1.00)	0.857
5 years	0.89 (0.79, 1.00)	0.89 (0.79, 1.00)	0.829
10 years	0.79 (0.26, 0.90)	0.79 (0.70, 1.00)	0.212

## Discussion

Long-term follow-up after ten years demonstrated in the present study similar patient-reported outcome measures and revision rates in CAS and conventional TKA.

The overall all-cause revision risk was 5% for all patients which is consistent with results of major arthroplasty registries [22–24]. There was no significant difference in the revision rates between both groups, which is consistent with most long-term studies comparing CAS and conventional TKA (Table 3). There is only one prospective study which demonstrated superior survival of CAS TKA [14]. This study included the learning curve as the surgeon had no prior experience with TKA and the results may therefore not be applicable to the majority of arthroplasty surgeons. Another study from the Australian registry demonstrated significant fewer revisions in patients aged less than 65 years at the time of surgery [13]. This might be one reason that the use of Computer-navigation in Australia has increased distinctly and was 33.5% in the 2018 report. This advantage of CAS TKA in the younger patients still existed in the latest report [24] (cumulative risk of revision in patients <65 years at 10/15 years: CAS 6.6% / 9.6%, conventional 7.7% / 11.1%). There was no difference in patients aged 65 years and older. In other prospective studies [25–29], revision rates were mostly higher in the conventional group although not statistically significant, which might be due to the limited number of patients. In two retrospective studies [15, 16], with a larger number of patients there was a significant better survival in the CAS TKA group. However, in both studies only less than 50% of the patients have been followed until ten years, which together with the retrospective design limits the validity of these data considerably.

**Table 3:** Summary of studies comparing CAS and conventional TKA with long-term follow-up

Study	Design	Age at surgery	Follow-up	Number of knees		Results
				Initially (follow-up)		
				CAS	Conv.	
Baier 2017 [16]	Matched pair	75y	10y	157 (75)	188 (75)	survival: CAS 98.1%, Conv. 92.5% (p=0.04), no difference in KSS and total WOMAC scores (WOMAC stiffness better in conv.)
Baumbach 2016 [15]	Retrospective	74y / 69y	10y	113 (50)	104 (46)	survival: CAS 98%, Conv. 87% (p<0.05), better alignment in CAS, no differences in HSS, KSS and SF-36 scores
Cip 2018 [25]	RCT	67y	12y	100 (47/32)*	100 (54/27)*	survival: CAS 98.2%, Conv. 91.5% (n.s.), no difference in KSS, HSS and WOMAC scores *analysis of survival / clinical follow-up
D’Amato 2018 [26]	RCT	69y	10y	60 (48)	60 (45)	survival: CAS 96.2%, Conv. 94.3% (n.s.) no differences in leg alignment, KSS and KOOS scores
De Steiger 2015 [13]	National registry	n.a.	9y+	44 573	270 545	survival all patients: CAS 95.4%, Conv. 94.8% (n.s.), survival <65y: CAS 93.7%, Conv. 92.2% (p=0.011)
Kim 2017 [27]	prospective	68y	12y	176 (162)	176 (162)	100% survival in both groups, no difference in KSS and WOMAC scores
Lacko 2018 [14]	RCT	67y	11y	30	31	survival: CAS 96.7%, Conv. 87% (p=0.04), alignment significantly better in CAS in a single-surgeon series without prior experience in TKA
Quanezar 2016 [28]	Institutional registry	70y	10y	87 (59)	51 (36)	survival: CAS 94%, Conv. 86% (n.s.),no difference in mechanical axis and KSS score
Song 2016 [29]	RCT	66y	9y	45 (41)	43 (40)	survival: CAS 100%, Conv. 95.3 % (n.s.), CAS had fewer alignment outliers (7.3 vs 20 %, p = 0.006), no difference in HSS, WOMAC, KS function and pain scores
Present study	RCT	66y / 69y	10y	40 (21)	40 (29)	Survival: CAS 92.5%, Conv. 97.5% (n.s.), no differences in KSS and EQ5D

Functional outcome measured with the Knee Society Score was not different between both groups at any follow-up in this study. This might be influenced by the fact that the aimed mechanical alignment was similar in both groups. It is, however, consistent with all other prospective studies with long-term follow-up [25–29]. Health-related quality of life demonstrated lower improvement but equivalent values when compared to an age-adapted standard population [30]. There were no differences between both groups in the present study.

Patient-reported outcome after TKA depends on several additional factors including rotational alignment, soft tissue-balance, the patello-femoral joint and especially patient-specific factors which might be even more important than leg axis and implant alignment. CAS TKA does not result in better rotational alignment [12] and it is not known whether the use of computer-navigation may influence patellar tracking. Several studies demonstrated that soft-tissue balancing can be more accurate and effective in CAS TKA [31–34]. This could not be investigated in detail in this study because a measured resection technique was used. However, patient-specific factors may have an even stronger correlation with functional results than intraoperative data which may be positively influenced by the use of a navigation system (alignment, ligament balance, range of motion) [35, 36]. Finally, TKA is a very effective procedure in terms of functional improvement and improvement in health-related quality of life. It is therefore difficult to further improve these results by any technology. Currently, despite better alignment, there is no evidence that Computer-navigation results in better patient-reported outcome measures.

The main limitation of this study is the relevant number of patients, which could not be followed until ten years after surgery. This is unfortunately common in these older patients. Only 50 from initially 80 patients (62.5%) were available for the 10-year follow-up, which limits the significance of the clinical results due to small group sizes.

## Conclusion

There was no difference between CAS and conventional TKA with regard to patient-reported outcome and revision risk ten years after surgery.

## Data Availability

The datasets generated during and/or analysed during the current study are not publicly available due to third party rights but are available from the corresponding author on reasonable request.

## Abbreviations

BMI: Body mass index
CAS: Computer-assisted navigation
EQ-5D: EuroQuol questionnaire
HrQoL: Health-related quality of life
KSS: Knee Society Score
PRO: Patient-reported Outcome
TKA: Total knee arthroplasty
VAS: Visual analogue scale
